# Characterization inference based on joint-optimization of multi-layer semantics and deep fusion matching network

**DOI:** 10.7717/peerj-cs.908

**Published:** 2022-04-12

**Authors:** Wenfeng Zheng, Lirong Yin

**Affiliations:** 1 School of Automation, University of Electronic Science and Technology of China, Chengdu, China; 2 Department of Geography and Anthropology, Louisiana State University and Agricultural and Mechanical College, Baton Rouge, Louisiana, United States

**Keywords:** Joint-Optimization of Multi-layer semantics, Deep fusion matching network, Meta-learning, Characterization inference, Natural language reasoning

## Abstract

The whole sentence representation reasoning process simultaneously comprises a sentence representation module and a semantic reasoning module. This paper combines the multi-layer semantic representation network with the deep fusion matching network to solve the limitations of only considering a sentence representation module or a reasoning model. It proposes a joint optimization method based on multi-layer semantics called the Semantic Fusion Deep Matching Network (SCF-DMN) to explore the influence of sentence representation and reasoning models on reasoning performance. Experiments on text entailment recognition tasks show that the joint optimization representation reasoning method performs better than the existing methods. The sentence representation optimization module and the improved optimization reasoning model can promote reasoning performance when used individually. However, the optimization of the reasoning model has a more significant impact on the final reasoning results. Furthermore, after comparing each module’s performance, there is a mutual constraint between the sentence representation module and the reasoning model. This condition restricts overall performance, resulting in no linear superposition of reasoning performance. Overall, by comparing the proposed methods with other existed methods that are tested using the same database, the proposed method solves the lack of in-depth interactive information and interpretability in the model design which would be inspirational for future improving and studying of natural language reasoning.

## Introduction

Natural language inference (NLI) has become one of the essential benchmark tasks in natural language understanding because of its complex language understanding and in-depth information involved in reasoning (Mykowiecka, 1991). Natural language reasoning technology is widely used in automatic reasoning (Van Harmelen, Lifschitz & Porter, 2008), machine translation (Macherey, Och & Ney, 2001), question answering systems (Hirschman & Gaizauskas, 2001), and large-scale content analysis. For example, Watson Q & A system designed and developed by IBM has defeated human champions in TV Q & A programs. Siri, a personal assistant, developed by Apple, has become the most successful voice assistant to help users realize a convenient life (Ma et al., 2021; Zheng et al., 2021a, 2021b). Other companies have also released personal intelligence assistants. The 836 National College Entrance Examination in 2017 was led by iFLYTEK (Liu, 2017). The examination’s mathematics test results show a certain gap between robots and humans in semantic understanding and logical reasoning. Compared with computer vision and speech recognition technology, natural language reasoning has not reached a high level because of its technical difficulties and complex application scenarios. It can be predicted that once the natural language reasoning technology makes a breakthrough and realizes the real barrier-free communication between humans and machines, the quality of human life will be significantly improved.

With the development of neural networks and deep learning, the sentence representation reasoning method has gradually developed from the traditional manual feature extraction method combined with the logical relationship reasoning method to the current end-to-end sentence representation reasoning method (Wang & Jiang, 2016; Zheng, Liu & Yin, 2021). The end-to-end sentence representation reasoning method overcomes the traditional method’s problems and shows a better performance and accuracy. As a result, it is widely used in various natural language processing tasks. There are two main research directions, namely, the sentence representation method and the semantic reasoning model under the natural language processing tasks.

With deep learning in natural language processing, semantic reasoning technology gradually changes from logic-based to deep learning-based reasoning technology (Ni et al., 2019). The core of reasoning technology based on deep learning calculates the similarity of two semantic objects. It simulates the potential correspondence between different abstract levels and different properties of semantic objects.

Bowman et al. (2015) proposed a deep reasoning framework for the first time. The potential relationship between text pairs is output after reasoning learning and classification processing at the inference network layer (recursive recurrent network). Wan et al. (2016) improved the inference network based on Bowman and proposed the MV-LSTM model, which is different from the deep reasoning framework. The interaction between semantic information is modeled as local reasoning information and the sentence expression order.

Hu et al. (2014) proposed a reasoning model based on a convolutional neural network (CNN), which has been successful in image processing, to natural language reasoning. The model is composed of an embedded layer and a convolution layer. Although the convolution structure has fixed depth characteristics, it will limit the combination of matching layers. However, network compensation can solve this limitation and realize the global comprehensive consideration of reasoning information. In the same year, Mou et al. (2016) proposed the TBCNN model, using tree convolution to combine sentence structure information into sentence representation. Finally, Bowman et al. (2016) proposed the stack-augmented parser-interpreter neural network (SPINN-PI model) to integrate tree structure sentence interpretation into the shift-reduce parser’s linear order structure.

Sun et al. (2017) proposed a reasoning model based on sentence coding. First, the internal attention mechanism was introduced into the bidirectional long-short memory network. Then, the first sentence code was used to replace the words appearing in the sentence. Due to the effectiveness of the “internal attention” mechanism, the model’s performance was significantly improved.

Influenced by the seq2seq model in machine translation, Zhao, Huang & Ma (2017) proposes a sentence representation reasoning model based on matching. The matching layer (inference layer) uses the attention mechanism to calculate the influence of each word in the premise sentence and each word in the hypothetical sentence. This layer first establishes the matching relationship between words and then obtains the matching relationship between sentence pairs. The sentence representation is reprocessed in the code layer. Finally, the highest matching degree between sentence pairs based on relation classification is selected in the hidden layer to realize sentence interactive matching reasoning.

Wang & Jiang (2016) proposed an improved attention-weighted model, match LSTM, based on Rocktäschel et al. (2015). In contrast to Rocktèschel’s emphasis on matching relations between sentences, match LSTM emphasizes more important word matching results. Experimental results show that this model is more conducive to predicting contradictory or neutral labels between sentences. Like Wang & Jiang (2016), Sha et al. (2016) designed a new LSTM unit, which takes the attention vector as an internal state and interacts with another sentence’s internal attention vector. Finally, Liu et al. (2016) proposed a model that pays more attention to the interaction of text pairs. Although it is helpful for the model’s reasoning ability, the modeling information is limited to shallow interactive information, which cannot simulate the complex reasoning process between sentences.

Parikh et al. (2016) proposed a more in-depth matching method for solving this problem, DecAtt. This problem divides the sentence pair reasoning task into several separate small tasks. It improves the reasoning depth by increasing the number of reasoning. Furthermore, Duan et al. (2018) proposed a deep matching network reasoning model (AF-DMN). AF-DMN uses a deep learning network to realize the extraction and fusion of deep semantics (such as sentences’ emotional information). Although it has good performance in capturing in-depth information, due to deep learning characteristics, compared with the DecAtt method, the whole reasoning process lacks interpretability.

The construction and optimization of sentence semantic representation and reasoning models have become the two core problems in sentence representation reasoning (Ni et al., 2019). No matter which aspect is improved, the whole sentence representation reasoning method’s effect will be affected. At the same time, it is of great significance to study the influence of the two methods on the sentence representation reasoning method.

This paper will further improve and optimize the existing sentence representation reasoning methods. This paper aims to solve the incomplete and inaccurate sentence semantic information expression, the inference model’s lack of in-depth interactive information, and the lack of interpretability during the reasoning process. The whole sentence representation reasoning process simultaneously comprises a sentence representation module and a semantic reasoning module. Therefore, this paper comprehensively considers the two modules’ improvement and optimization, combining the multi-layer semantic representation and deep fusion matching networks. Finally, it proposes a joint optimization sentence representation reasoning method to explore sentence representation and reasoning, learning the prediction results.

## Materials and Methods

In order to verify the feasibility of the semantic-based sentence representation reasoning method in the field of text entailment and to explore the influence of sentence representation and reasoning learning on sentence representation reasoning, experiments were carried out on SNLI data sets and Multi-NLI data sets commonly used in the field of text entailment. All the methods and experiment are based on python 2.7 based on pyTorch environment.

### SNLI dataset

SNLI data set is a text entailment data set published by Stanford University. SNLI is manually annotated and contains 570k text pairs. There are three kinds of marks: entailment, contradiction, and neutral. In this paper, data are divided into a training set (549,367 samples), a verification set (9,842 samples), and a test set (9,824 samples) according to Zhu et al. (2018) data partition rules. Some SNLI data forms are shown in Table 1.

**Table 1:** Sample data of SNLI dataset.

Premise sentence	Label	Hypothetical sentence
Two women are embracing while holding to-go packages	Entailment E E E E E	Two women are holding packages
A man sold donuts to a customer during a world exhibition event held in the city of Angeles	Contradiction C C C C C	A woman drinks her coffee in a small café
A man in a blue shirt is standing in front of a garage-like structure painted with geometric designs	Neutral N E N N N	A man is repainting a garage

### Multi-NLI dataset

The Multi-NLI dataset (Williams, Nangia & Bowman, 2018) contains 433k text pairs, different from the SNLI dataset. It covers more data close to real life, such as novels and letters. The sample data is shown in Table 2. The data set contains 10 categories of data. The same category appears in the training and test sets simultaneously. It is divided into matched and mismatched sets.

**Table 2:** Sample data of Multi-NLI dataset.

Categories	Premise sentence	Label	Hypothetical sentence
Novels	The Old One always comforted Ca’daan, except today	Neutral	Ca’daan knew the Old One very well
Letters	Your gift is appreciated by every student who will benefit from your generosity	Neutral	Hundreds of students will benefit from your generosity
Telephone	Yes, now you know if everybody like in August when everybody’s on vacation or something we can dress a little more casual or	Contradiction	August is a blackout month for vacations in the company

In this paper, the text entailment task is carried out on the unmatched and matching sets. The data are divided into the training set (392,702 samples) and the matching/mismatching verification set (9,815/9,832 samples).

### Method

#### Parameter setting

All experiments are based on the Theano deep learning framework. The NVIDIA GeForce GTX 1070 graphics card and 16 g memory train and test the model performance. The experimental parameters are as follows:

1. The word vector pre-training is initialized by GloVe-840B-300D (Pennington, Socher & Manning, 2014), in which the word embedding vector dimension is 300D. For the words not in the vocabulary, Gaussian distribution is used for random initial vectorization, and the character vector corresponding to the word is updated with the training process;
2. The gradient update of the model adopts Adam (Kingma & Ba, 2015) algorithm and its default parameters ${\alpha _1}$ and ${\alpha _2}$ are 9 and 0.99 respectively. ReLU function was used in all activation functions of the model. Both function and algorithm are selected for their outstanding performance when applied to neural networks.
3. The training process adopts the early stop strategy and combines dropout and data batching to avoid model overfitting. Dropout is set to 0.8. For the SNLI dataset, each batch contains 32 sentence pairs, and for the Multi-NLI dataset, each training batch contains 8 sentence pairs.
4. The learning rate is initialized to 0.0002. After each iteration, the model uses the results of the verification set to adjust the learning parameters. For example, when the verification set’s accuracy decreases, the learning rate is reduced by half, and the early stop flag is increased by one.

#### Joint method design based on multi-layer semantics and SCF-DMN

The existing research only considers the optimization and promotion of sentence representation module or semantic reasoning model. Suppose the two modules are optimized at the same time. In that case, the limitation of single optimization can be solved to a certain extent. However, there is no research content to consider the joint optimization of the two modules simultaneously. Therefore, this paper combines the multi-layer semantic representation network and deep fusion matching model to form the sentence representation optimization methods. The joint optimization method consists of four modules: preprocessing, text representation, relational reasoning, and result prediction

1. Preprocessing: preprocessing the premise text P and hypothesis text H by word segmentation and part of speech restoration to generate the premise hypothesis text pair.
2. Text representation: a multi-layer semantic representation network is used to obtain the sentence’s semantic representation of premise text P and hypothesis text H, respectively.
3. Relational reasoning: deep fusion matching network is used to infer the possible score of implication relationship between premise text P and hypothesis text H.
4. Result prediction: the score of implication relation is classified. The result with the highest possibility is output as the result of the relational reasoning system.

A. Preprocessing

The first step is the natural language text preprocessing. The input pairs are all English texts, so the preprocessing includes the English word segmentation, speech restoration, removing stop words, and processing non-standard numbers. The word is the smallest language unit in the natural language reasoning task, so segmenting the input text before sentence representation is necessary. Stop words generally refer to the words which appear frequently but have no practical significance compared with other words in sentences. In this paper, to save storage space and improve efficiency, stop words are removed. In this paper, all the contents related to numbers are unified into Arabic numerals.

B. Sentence representation

After the preprocess module, the next module in the process is the sentence representation module. Itis based on the multi-layer semantic representation network. The sentence’s semantic representation of the text vector of premise text P and hypothesis text h is carried out. The specific operation is as follows:

First of all, all words in the question $H = ({H_1},{H_2},...,{H_n})$ are vectorized. Then, the word vector expression at the word level is obtained through the seq2word module of multi-layer semantic representation network; then the word vector expression of character level is obtained by word2char model. The embedded coding ${S_H}$ of hypothetical text is obtained by splicing operation.

Embedding encoding ${S_H}{\rm \; }$ of text input, the coding layer of the multi-layer semantic representation network captures the semantic information of different levels of the hypothesis test. The layer then outputs the complete sentence representation $S_H^{\rm '}$ of the hypothesis text after fusion.

Like the hypothesis text, the premise text P is input into the multi-layer semantic representation network with the same configuration. The sentence representation of the premise text is obtained as follows. The number of layers and word order information weight alpha of the multi-layer semantic representation network is adjusted according to the model’s training results.

C. Relational reasoning

The third module would be the relational reasoning module. The semantic fusion deep matching network (SCF-DMN) is used in the relational reasoning module to obtain inference between premise text P and hypothesis text h based on the text pair’s embedded representation. The relational reasoning module mainly includes the convolution layer, matching layer, and information aggregation layer.

1. Convolution layer

**Figure 1:**
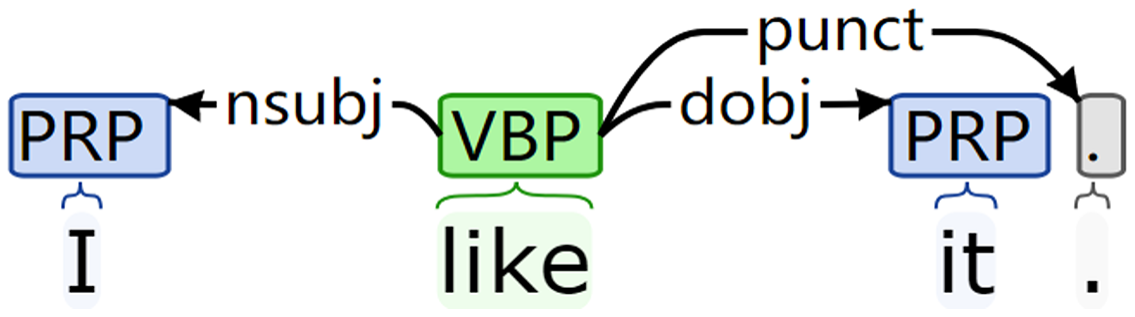
Visualization results of the dependency syntax tree.

The text representation module obtains the embedded representation of premise hypothesis pairs but does not contain syntactic structure information. Therefore, this module’s convolution layer uses the dependency tree convolution network (d-TBCNN) to obtain the syntactic structure information of premise text P and hypothesis text H (Mou & Jin, 2018). The specific steps are as follows:

Firstly, the natural language parser in NLTK is used to transform the premise text P (hypothetical text H) into dependency syntax tree form. For example, the visual dependency tree structure is shown in Fig. 1.

Then, according to the sequence of dependent subtrees obtained, the convolution operation is performed on the dependency results of each subtree in turn. The convolution results of all subtrees are summarized to form the syntactic structure information ${T_P}$( ${T_H}$) of premise text P (hypothesis text H). For example, the sentence “I like it.” has three subtrees. The convolution sequence of subtrees with “I” as the parent node is as follows:

$I \to Conv\_I \to {\rm PoolLayer} \to {\rm hidden}$

$I \to Conv\_like \to {\rm PoolLayer} \to {\rm hidden}$

The subtree contains two directly connected nodes, “I” and “like.” All nodes put forward the syntactic structure information carried by the convolution layer. The convolution result of the subtree went through the pooling layer and the hidden layer and are summarized to generate the structure information corresponding to the sentence.

2. Matching layer

The matching layer adopts the chain structure, composed of M matching modules with the same configuration to collect local reasoning information based on the sequence. The specific calculation steps are as follows:

1) Get interactive information

For section i-th matching module, the cross attention between the premise hypothesis pairs is calculated, and then the cross attention is used to weigh the text pair embed representation ( $S_P^{\rm '},S_H^{\rm '}$) and generate the premise-hypothesis interaction information $A_{P - H}^i{\rm \; \; }$of the layer and hypothesis-premise interaction information $A_{H - P}^i$.

2) Get self-interested information

For the generated premise-hypothesis interaction information $A_{P - H}^i$ and hypothesis-premise interaction information $A_{H - P}^i$, the corresponding self-attention information ${A^\prime}_{P - H}^i$ and ${A^\prime}_{H - P}^i$ were calculated respectively and to strengthen interactive information on the semantic information of the text itself.

3) Information fusion

The interactive information and self-attention information generated in step 1) and step 2) are fused to form the reasoning information $M_{P - H}^i = \left( {A_{P - H}^i,{A^\prime}_{P - H}^i} \right)$ and $M_{H - P}^i = \left( {A_{H - P}^i,{A^\prime}_{H - P}^i} \right)$ of the matching module.

4) Generating local reasoning information

Along the chain structure, based on the reasoning information generated by the previous matching module, repeat steps 1–3 m times, and finally take the output of the top-level as the output of the matching layer, the premise text matching semantics ${M_{P - H}}{\rm \; }$and hypothetical text matching semantics ${M_{H - P}}$.

3. Information aggregation layer

The information aggregation layer is used to construct the input of inference prediction layer fuse the text syntactic structure features ${T_P}$ and ${T_H}$ obtained by convolution layer and the local reasoning information ${M_{P - H}}$, ${M_{H - P}}$ obtained from the and matching layer. Finally, a fixed size text fusion reasoning information ${F_P} = \left( {{T_P},{M_{P - H}}} \right)$ and ${F_H} = \left( {{T_H},{M_{H - P}}} \right)$ is generated. The fusion method uses the control gate composed of the Sigmod function and dot multiplication operation. It adjusts the fusion weight by learning the importance of syntactic structure and local reasoning information for relational reasoning.

D. Result prediction

The final module is the result prediction module. The inference and prediction layer comprises three layers of the fully connected neural network. The output of the information aggregation layer passes through a single hidden layer and activation function. Finally, it classifies by the Softmax function to obtain the probability of implication relationship between current text pairs. The output result module selects the most probable result according to the probability of implication relation.

### Design of experimental scheme

In order to explore the influence of sentence representation module and relational reasoning model on reasoning results, four groups of experimental schemes were designed to compare and analyze the performance of sentence representation module and relational reasoning model without optimization, single optimization, combinatorial optimization, and joint optimization, to understand the interaction between modules.

1) No optimization

**Figure 2:**
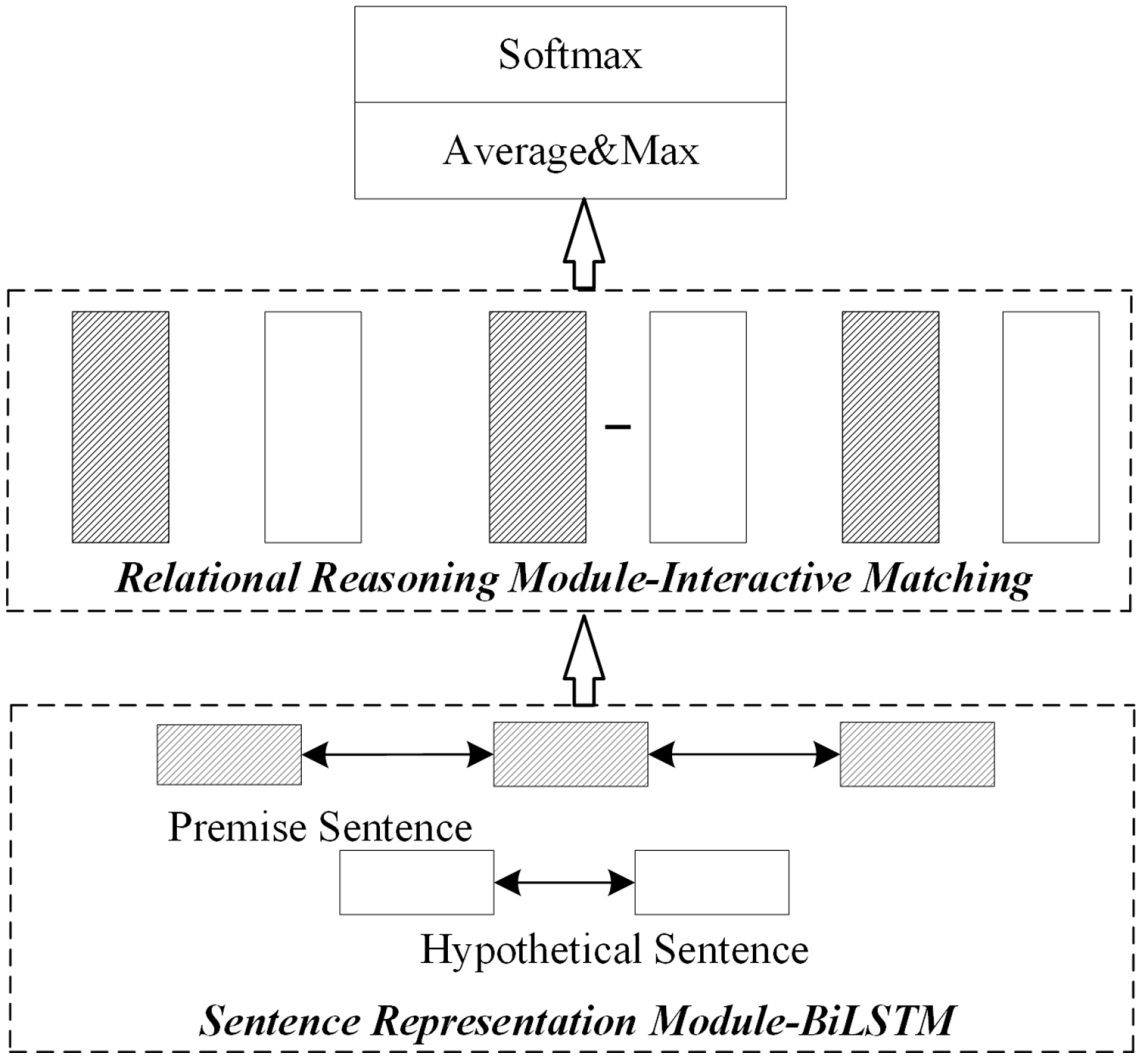
Network structure without module optimization.

This scheme does not consider the optimization of the sentence representation module or relational reasoning module. Instead, this paper uses the general sentence representation reasoning method proposed by Graves, Jaitly & Mohamed, 2013 as the non-optimization model. As shown in Fig. 2, after the preprocess, the sentence representation module uses the bidirectional long-short memory network to obtain the sentence embedding representation. The relational reasoning module uses the interactive matching method to obtain interactive information between sentences. Finally, it obtains the probability distribution result of implication relation through pooling and full connection layers (Ding et al., 2019; Graves, Jaitly & Mohamed, 2013).

2) Separate optimization

The single optimization experiment only optimizes the sentence representation module or the relational reasoning model. The non-optimized module is consistent with the corresponding module in the non-optimization experimental scheme. The single optimization experiment’s result prediction module uses average pooling and maximum pooling to fix the sentence pair vector’s length. It then obtains the final implication through the full connection layer and Softmax functions. The single optimization experiment includes two parts

1. Optimizing sentence representation module separately

**Figure 3:**
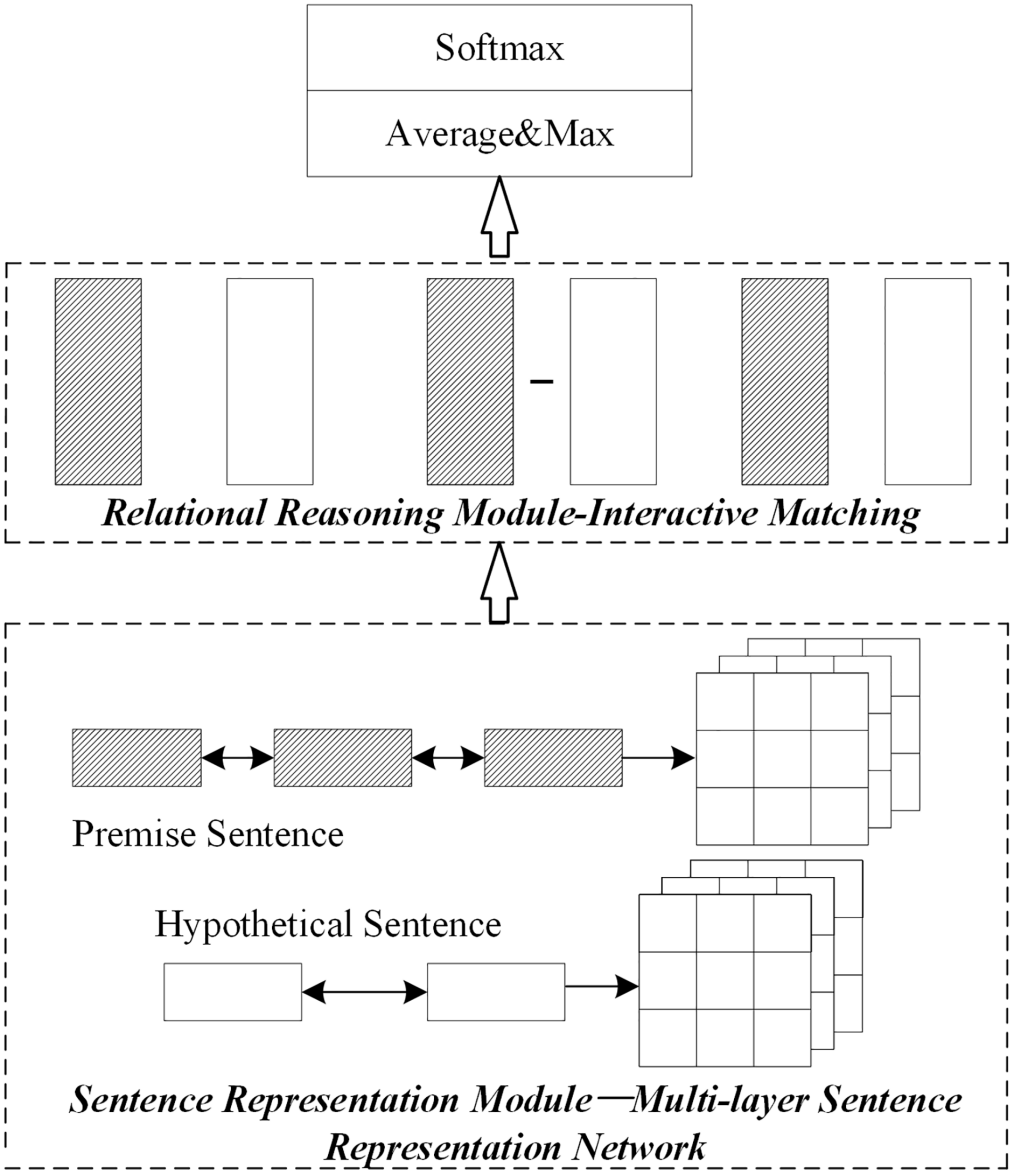
Network structure diagram of sentence representation module optimization.

Figure 3 shows the basic structure of the optimized sentence representation module. The initial value of the semantic layer level is set to 5. In addition, the initial weight parameter alpha of word order information is set to 1.5; other parts’ parameters are set with general parameters.

2. Single optimization of relational reasoning module

**Figure 4:**
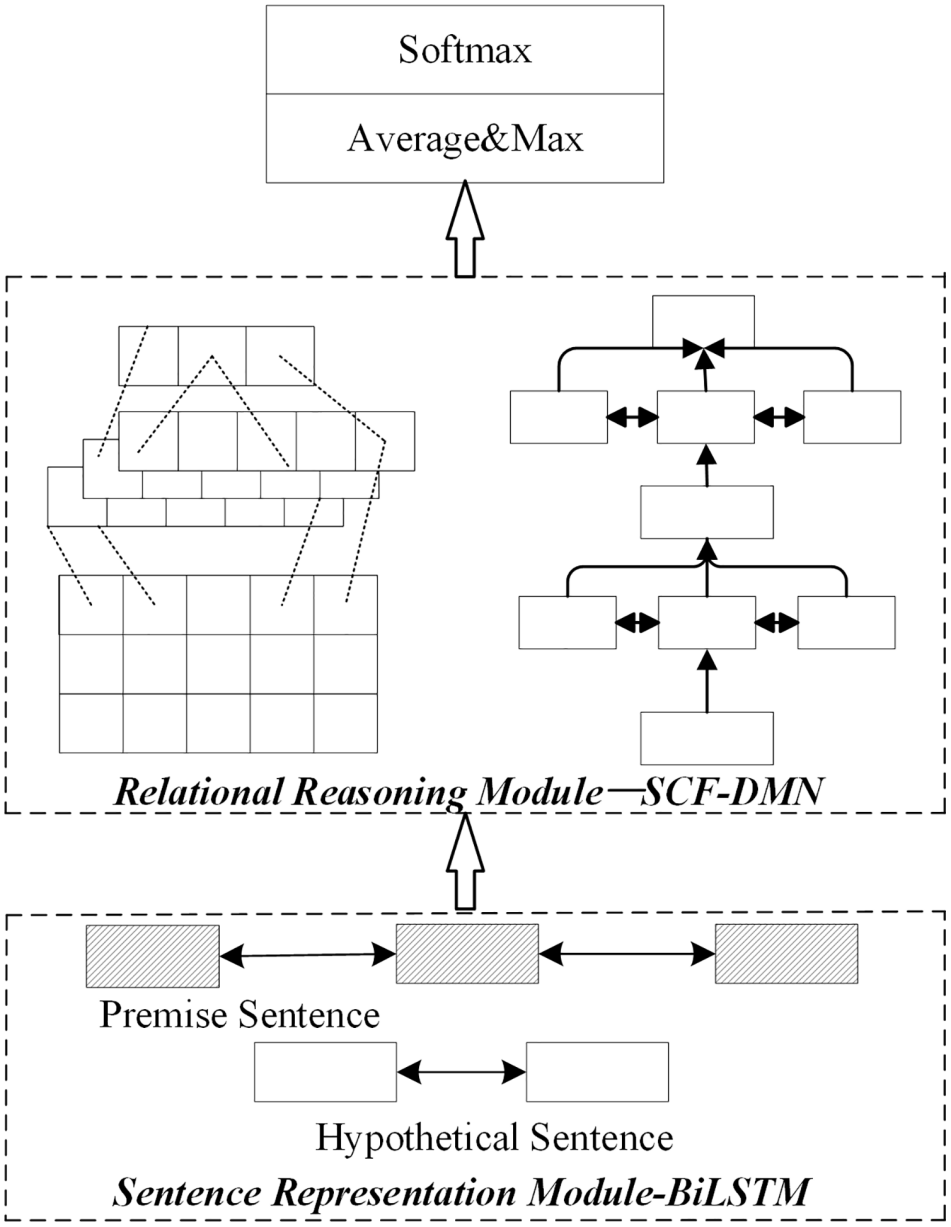
Network structure diagram of relation reasoning module optimized separately.

The network structure diagram of the single optimized relational reasoning module is shown in Fig. 4. First, the sentence representation module uses the bidirectional long-short memory network to obtain the embedded representation. Next, the deep fusion matching network is used to optimize the relational reasoning module. Next, the local reasoning information and syntactic structure information between sentence pairs are obtained based on the sentence embedding representation. Finally, based on the obtained reasoning, information predicts the implicative relationship between sentence pairs.

For the SNLI dataset, the number of matching modules in the matching layer is set to 3; for the number of Multi-NLI matching modules, T is set to 2. The parameters of other parts are set with general parameters. The initial numbers for modules are chosen based on the number used by other studies using similar networks on the same datasets.

3) Combination optimization

The combination optimization of the sentence representation module and the relational reasoning model is realized by taking the average output probability distribution of the two optimization modules’ implication relation as the final probability distribution.

**Figure 5:**
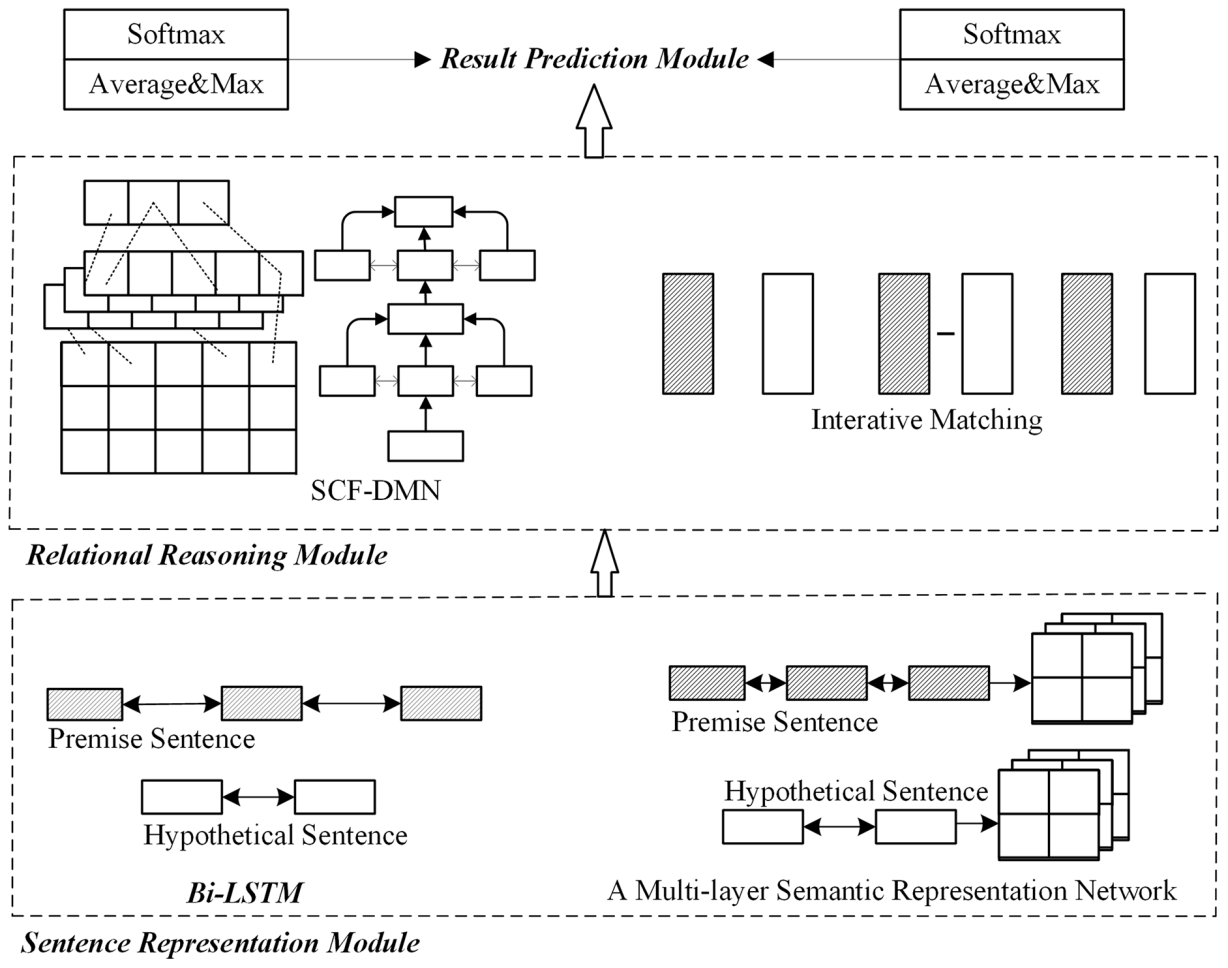
Network structure of combined optimization scheme.

The combination optimization model’s network structure is shown in Fig. 5, mainly divided into two parts. The left side is the sentence representation reasoning method, which optimizes the relationship reasoning module alone. The right side is the sentence representation reasoning method, which optimizes the sentence representation module separately. The specific steps of the combination optimization experiment are as follows:

Firstly, the left SCF-DMN network is trained. Then, the bidirectional long-short memory network learns the sentence semantics and context information. The interactive information is obtained by using the deep fusion matching network designed in this paper. After pooling and classification functions, the left network’s implication relationship’s probability distribution results are obtained.

The multi-layer semantic representation network on the right is trained synchronously. The multi-layer semantic representation network designed in this paper is used to capture multiple sentences’ semantic information. The inference information between sentences is obtained by interactive matching. Finally, the probability distribution results of this part’s implication relation are obtained through average pooling, maximum pooling, and full connection layer.

Finally, the average output probability distribution of the left and right networks is taken as the final result of the combination optimization scheme.

The parameters in the combination optimization scheme are updated and learned synchronously with the training process. All neural network dimensions are set to 300D. The number of initial matching modules is set to 2. The initial semantic layer level is 5. The initial weight parameter alpha of word order information is set to 1.5. The remaining network parameters are randomly initialized by the Gaussian function (Glorot & Bengio, 2010).

4) Joint optimization

**Figure 6:**
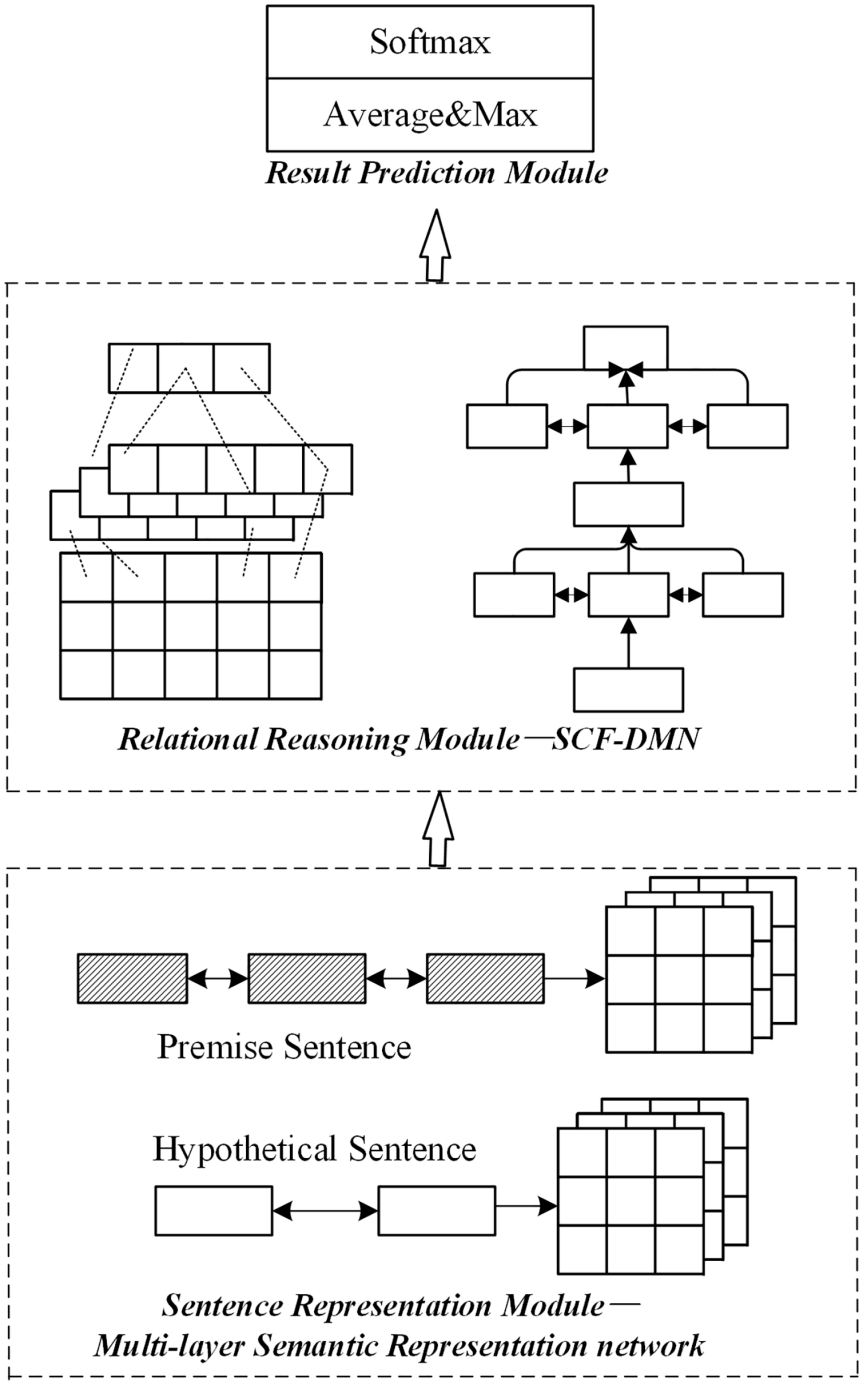
Network structure of joint optimization scheme.

The joint optimization scheme of the sentence representation module and relation module is the joint optimization method based on the multi-layer semantic representation network and deep fusion matching reasoning model proposed in this paper. The joint optimization method is shown in Fig. 6.

The sentence representation module adopts the multi-layer semantic representation network in this paper in the joint optimization scheme. The relational reasoning model adopts the deep fusion matching network designed in this paper. Firstly, a complete sentence embedding representation is obtained through the multi-layer semantic representation network. On this basis, the local reasoning information and syntactic structure information between sentence pairs are captured through the deep fusion matching network. Finally, the implicative relation between sentence pairs is obtained by pool level and full connection layer reasoning.

## Results

### Experimental results on SNLI dataset

The first mock exam results on the SNLI dataset are shown in Table 3. The joint and combinatorial optimization schemes on SNLI datasets are significantly better than a single module. The accuracy of training programs and test sets is increased by 4.7% and 3.3%, respectively. The results show that optimizing the sentence representation module and inference module simultaneously improves the sentence representation reasoning method’s performance. The promotion effect between the two modules is mutual.

**Table 3:** Accuracy of each optimization scheme on the SNLI dataset.

Program	Training set (%)	Test set%
300D Tree-CNN (Mou et al., 2016)	83.3	82.1
300D NSE (Munkhdalai & Yu, 2017)	86.2	84.6
100D LSTMs with attention (Rocktäschel et al., 2015)	85.3	83.5
100D Deep Fusion LSTM (Liu et al., 2016)	85.2	84.6
300D Matching-LSTM (Wang & Jiang, 2016)	92.0	86.1
200D Decomposable Attention Models (Parikh et al., 2016)	90.5	86.8
300D Re-read LSTM (Sha et al., 2016)	90.7	87.5
600D ESIM (Ni et al., 2017)	92.6	88.0
AF-DMN (Duan et al., 2018)	94.5	88.6
Modular optimization (This paper)	90.4	85.0
Sentence representation module optimized (This paper)	91.7	86.1
The relational reasoning module optimized (This paper)	95.8	89.0
Combinatorial optimization (This paper)	96.4	89.4
Joint optimization (This paper)	96.2	88.8

Compared with the combined optimization scheme’s experimental results and the joint optimization scheme, the combination optimization scheme’s accuracy is higher in both sets. The accuracies are 96.4% and 89.4%, respectively. Thus, the results show that the sentence representation module and reasoning module as a joint optimization method of the overall model training optimization can improve sentences’ reasoning performance. However, the improvement effect is not as good as the combination optimization scheme formed by linear superposition.

It is speculated that this result is the mutual restriction between sentence representation and reasoning model. When all parameters are trained as a whole, each part cannot achieve the best performance. Thus, although the overall performance is improved, there is no expected substantial improvement.

As shown in Table 3, there are the comparison of the proposed four methods and some already existed methods that are trained and tested using the SNLI dataset. The methods proposed by this paper has a higher accuracy in both training and test sets comparing to the existing methods. The highest accuracy belongs to the combinatorial optimization method. The joint optimization method has a lower accuracy (88.8%) on the test set compare to the combinatorial optimization (89.4%) and the relational reasoning module optimized (89.0%) methods.

### Experimental results on Multi-NLI dataset

Table 4 shows the accuracy of each optimization scheme on the Multi-NLI dataset. The joint optimization scheme, which combines a multi-layer semantic network and a deep fusion matching network, has an accuracy rate of 77.3% on the Multi-NLI matching set. The result is better than the modular module independent optimization scheme. The accuracy rate on the non-matching set is 75.1%, which is close to but slightly lower than that of the single optimized relational reasoning module.

**Table 4:** Accuracy of each model on the Multi-NLI dataset.

Program	Matching set%	Unmatched set (%)
CBOW (Williams, Nangia & Bowman, 2018)	64.8	64.5
BiLSTM (Williams, Nangia & Bowman, 2018)	66.9	66.9
ESIM (Ni et al., 2017)	76.8	75.8
AF-DMN (Duan et al., 2018)	76.9	76.3
Modular optimization	66.9	66.9
Sentence representation module optimized	73.6	73.8
The relational reasoning module is optimized	77.1	75.3
Combinatorial optimization	77.5	75.5
Joint optimization	77.3	75.1

The Multi-NLI data set’s experimental results show that the sentence representation reasoning method’s performance is affected by both sentence representation and the reasoning model. Therefore, the optimization of both helps to improve reasoning performance. However, combining the two parts is not good when the two modules are optimized jointly due to the interaction between the two modules.

Table 4 also shows some of the benchmark methods that are trained and tested using the Multi-NLI dataset. The combinatorial optimization method has the highest accuracy among the five proposed methods and has the highest accuracy rate on the matching set and the second highest accuracy rate on the unmatched set. The combinatorial optimization (77.5%), the joint optimization (77.3%), and the optimized only relational reasoning module (77.1%) methods all show a higher accuracy on matching set compare to the AF-DMN method (76.9%), but they have a lower accuracy on the unmatched set (75.5%, 75.1%, 75.3%) comparing to the AF-DMN method (76.3%). Through the difference are slight since all these methods performance well on the Multi-NLI dataset, the reason behind these difference will be important for the further improvement of the model.

## Discussion

### Analysis of optimization mode

Two groups of comparative experiments were conducted to explore further the influence of the optimization model of sentence representation module and reasoning learning module on reasoning results. First, the influence of combinatorial and joint optimization on sentence representation reasoning and joint optimization and individual optimization of each module on reasoning performance are analyzed.

1. Comparison between joint optimization and combinatorial optimization

Table 5 shows the performance of the hybrid model on the SNLI dataset under different optimization methods. The accuracy rate of combined optimization is higher than that of joint optimization. However, the training time and the number of super parameters of joint optimization are less than that of combinatorial optimization, which is more in line with actual tasks’ real-time requirements.

**Table 5:** Comparison of the influence of different optimization methods on sentence representation reasoning method.

Optimization mode	Accuracy rate (%)	Training time (H)	Hyperparameters
Combinatorial optimization	89.4	32.87	49,196,195
Joint optimization	88.8	30.64	48,077,358

The experimental results show that the combination optimization of the sentence representation module and inference module to achieve the optimization and then take the average prediction value as the final result can weaken the restriction. However, the time-consuming and the number of parameters in the process increase significantly, improving the reasoning process’s complexity and reducing the interpretability.

**Figure 7:**
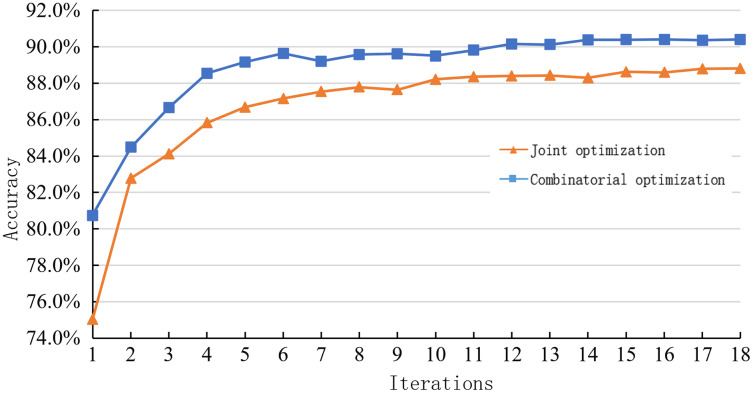
Learning curve of joint optimization and combination optimization on the SNLI dataset.

Figure 7 shows the learning curves of the sentence representation reasoning method using combinatorial and joint optimization methods on the SNLI dataset.

It can be observed from the figure that the combination optimization method has higher iteration times than the joint optimization method at the same time. The experimental results also show that the sentence representation module and the reasoning module’s optimization will be affected by the interaction between the two modules in the same model. Therefore, the performance cannot be improved linearly as the combination optimization.

2. Comparison of joint optimization and individual optimization of each module

**Figure 8:**
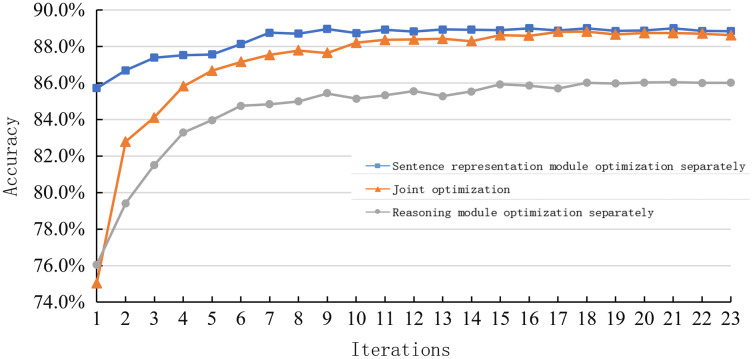
Learning curve of joint optimization and single optimization on the SNLI dataset.

Figure 8 shows the learning curve of a sentence representation method based on a multi-layer semantic representation network, a reasoning model based on a deep fusion matching network, and a joint sentence representation reasoning method based on multi-layer semantic and deep fusion matching network proposed in this paper on the SNLI dataset. Overall, the learning curve based on the joint optimization method is between the single optimization scheme of the sentence representation module and the reasoning module.

When the number of iterations is between 1–3, this model’s learning rate is close to the rate of the multi-layer semantic representation network. When the number of iterations is 4–15, this model’s changing trend is consistent with a deep fusion matching network. In the later stage of training, the learning curve matches that based on deep fusion.

This model’s learning curve (joint optimization) shows that sentence representation greatly influences the early reasoning ability of the model. On the other hand, the inference model strongly influences the reasoning ability in the middle and later stages of the model. Simultaneously, it is also found that the combination of sentence representation and reasoning methods can achieve better results because of their respective network structures. However, it is limited by the mutual constraints between the various parts, and there is no greater improvement.

### Performance change analysis of each module

By comparing different optimization methods, it is found that the sentence representation module and relational reasoning module are optimized simultaneously. Their interaction restricts the model’s performance improvement, but each module’s specific impact is unknown. Therefore, this paper further studies the impact of this interaction on each module. The specific experimental results and analysis are as follows.

1. The influence on sentence representation module

**Figure 9:**
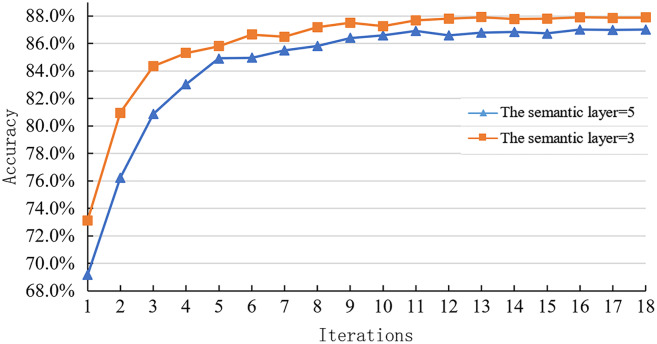
Learning curve of joint optimization model with different semantic levels on the SNLI dataset.

This paper experimented on the semantic layer level change of sentence representation module in the joint optimization scheme on SNLI dataset to explore the influence of inference module on sentence representation module. The experimental results are shown in Fig. 9.

Figure 9 shows the joint optimization model’s learning curve with different semantic layer levels on the SNLI test set. It can be observed that when the number of semantic layers is equal to 3, the accuracy of the model on the SNLI dataset is higher than that when the sentence representation module takes other semantic levels. Specifically, the number of semantic layers decreases from 5 to 3. As a result, the model’s accuracy increases from 87.0% to 87.87%, which is increased by 0.87%.

2. Influence on reasoning module

Similarly, this paper explores the impact of the interaction between the two on the inference module’s performance. The optimized inference module uses the deep fusion matching network, mainly affected by the number of matching layers and syntactic structure. Since the performance changes in the syntactic structure cannot directly reflect the model’s performance changes, this paper mainly explores the performance changes of the reasoning module by comparing and analyzing the learning curves of the reasoning models with different matching layer levels on the SNLI dataset.

**Figure 10:**
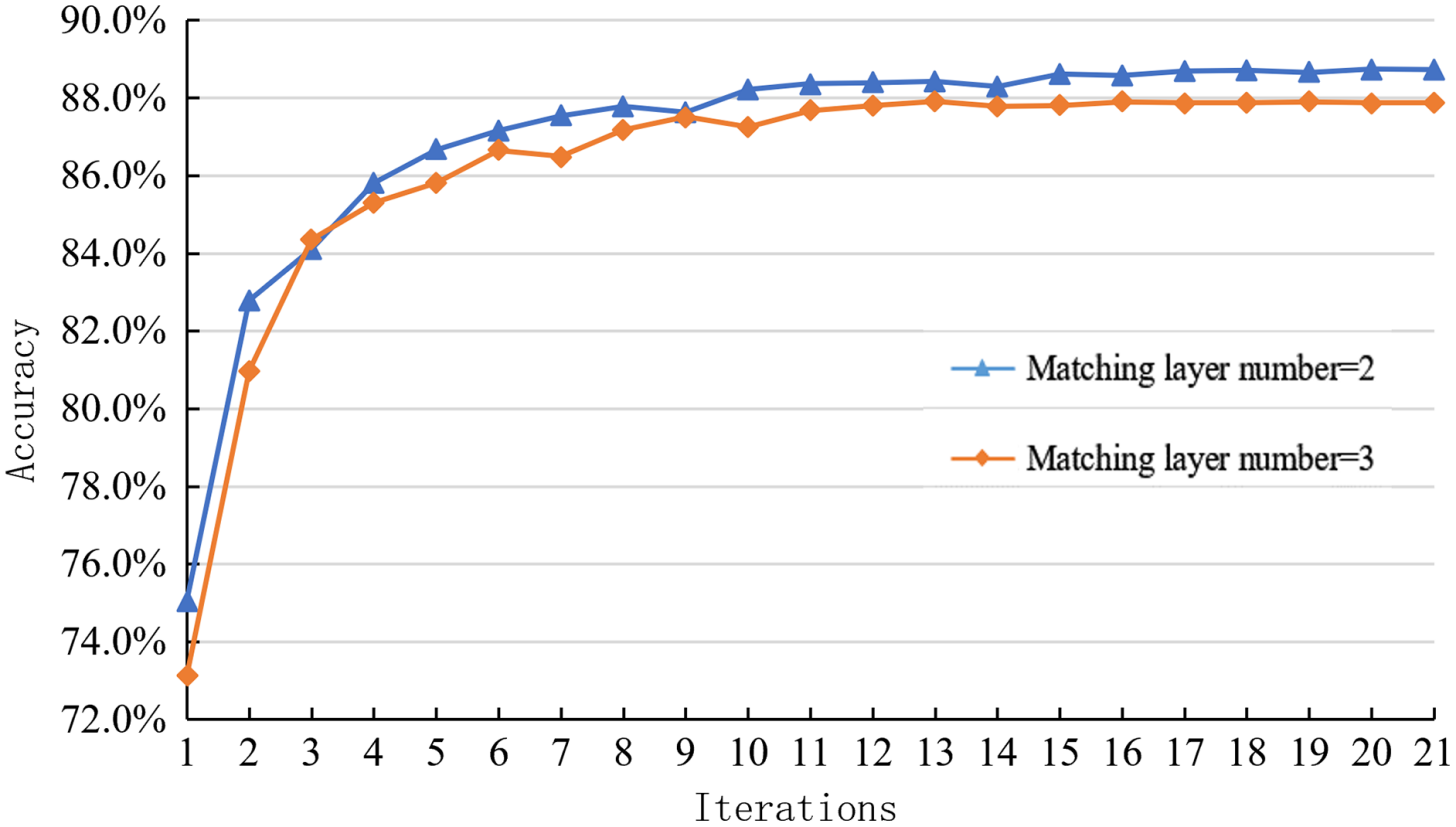
Learning curve of joint optimization model with different matching levels on the SNLI dataset.

As shown in Fig. 10, the joint model’s learning curve corresponding to different matching layer levels on the SNLI dataset can be seen. The optimal matching layer level of the joint optimization model is two layers. The optimal matching layer of the independent optimization reasoning model is three layers. After the combination, the number of matching layers decreases, but the reasoning accuracy on SNLI data increases from 87.8% to 88.8%, increasing by 1%. Therefore, the optimization of the sentence representation module also restricts the performance of the inference module.

Based on the experimental results of this part, it is found that the interaction between the sentence representation module and the reasoning model can improve the overall performance of the joint model. However, on the other hand, it will restrict the performance of each module.

## Conclusions

In this paper, considering the joint optimization of the sentence representation module and the relational reasoning module, a sentence representation reasoning method that combines a multi-layer semantic network and a deep fusion matching network is proposed. Specifically, firstly, the complete sentence embedding representation is obtained by fusing a multi-layer semantic network. Next, a deep fusion matching network obtains the local reasoning and syntactic structure information between sentence pairs. Then the reasoning results are obtained through the pooling layer and full connection layer. Finally, the performance of the joint optimization model is explored on several data sets containing recognition tasks. The joint optimization model’s advantages and disadvantages are compared with those without optimization, individual optimization, and combinatorial optimization. Experimental results show that the sentence reasoning method’s joint optimization has better accuracy than other existing characterization reasoning methods. However, comparing separate optimization sentence characterization and optimization effect between reasoning models, the optimization effect is closer. This result suggests that sentence representation and reasoning model optimization function have a larger influence on the final result of reasoning on promoting the reasoning and inference model of optimizing. After comparing the experimental results of joint optimization and combinatorial optimization, there are mutual constraints. These constraints restrict optimal performance, resulting in no linear superposition of reasoning performance after joint optimization.

At present, the field of natural language reasoning has become another research hotspot after the field of images. Many scholars have carried out research work in this field. However, sentence representational reasoning is still the focus and difficulty in this field due to cognition and understanding. Although this paper explores sentence representation and reasoning methods that improve reasoning accuracy, it is far from the best effect. Given this, future research can be further studied from the following contents:

This article explores the characterization of the sentence module and a reasoning model for sentences expressing the influence of reasoning methods and found mutual promotion and mutual constraints. However, this article does not put forward specific proposals for how to solve the constraints between them. Subsequent can consider solving the constraints between the two to realize partial performance improvement and improve the overall performance.

## Supplemental Information

10.7717/peerj-cs.908/supp-1 Supplemental Information 1 Code for the Joint-Optimization of Multi-layer semantics and Deep Fusion Matching Network.

## Additional Information and Declarations

### Data Availability

The following information was supplied regarding data availability: The raw data is available at: - The Stanford Natural Language Inference (SNLI) *Corpus*: https://nlp.stanford.edu/projects/snli/ - The Multi-Genre NLI *Corpus*: https://cims.nyu.edu/~sbowman/multinli/
